# Limited heat tolerance in an Arctic passerine: Thermoregulatory implications for cold‐specialized birds in a rapidly warming world

**DOI:** 10.1002/ece3.7141

**Published:** 2021-01-17

**Authors:** Ryan S. O'Connor, Audrey Le Pogam, Kevin G. Young, Francis Robitaille, Emily S. Choy, Oliver P. Love, Kyle H. Elliott, Anna L. Hargreaves, Dominique Berteaux, Andrew Tam, François Vézina

**Affiliations:** ^1^ Département de Biologie, Chimie et Géographie Université du Québec à Rimouski Rimouski QC Canada; ^2^ Groupe de recherche sur les environnements nordiques BORÉAS Rimouski Canada; ^3^ Centre d'études nordiques Rimouski Canada; ^4^ Centre de la science de la biodiversité du Québec Rimouski Canada; ^5^ Department of Biology Advanced Facility for Avian Research Western University London ON Canada; ^6^ Department of Natural Resource Sciences McGill University QC Canada; ^7^ Department of Integrative Biology University of Windsor Windsor ON Canada; ^8^ Department of Biological Sciences McGill University Montreal QC Canada; ^9^ Department of National Defence, 8 Wing Environment Astra ON Canada

**Keywords:** Arctic climate change, evaporative cooling efficiency, evaporative water loss, heat dissipation, snow bunting, thermal physiology, thermoregulatory polygon

## Abstract

1. Arctic animals inhabit some of the coldest environments on the planet and have evolved physiological mechanisms for minimizing heat loss under extreme cold. However, the Arctic is warming faster than the global average and how well Arctic animals tolerate even moderately high air temperatures (*T*_a_) is unknown.
2. Using flow‐through respirometry, we investigated the heat tolerance and evaporative cooling capacity of snow buntings (*Plectrophenax nivalis*; ≈31 g, *N* = 42), a cold specialist, Arctic songbird. We exposed buntings to increasing *T*_a_ and measured body temperature (*T*_b_), resting metabolic rate (RMR), rates of evaporative water loss (EWL), and evaporative cooling efficiency (the ratio of evaporative heat loss to metabolic heat production).
3. Buntings had an average (±*SD*) *T*_b_ of 41.3 ± 0.2°C at thermoneutral *T*_a_ and increased *T*_b_ to a maximum of 43.5 ± 0.3°C. Buntings started panting at *T*_a_ of 33.2 ± 1.7°C, with rapid increases in EWL starting at *T*_a_ = 34.6°C, meaning they experienced heat stress when air temperatures were well below their body temperature. Maximum rates of EWL were only 2.9× baseline rates at thermoneutral *T*_a_, a markedly lower increase than seen in more heat‐tolerant arid‐zone species (e.g., ≥4.7× baseline rates). Heat‐stressed buntings also had low evaporative cooling efficiencies, with 95% of individuals unable to evaporatively dissipate an amount of heat equivalent to their own metabolic heat production.
4. Our results suggest that buntings’ well‐developed cold tolerance may come at the cost of reduced heat tolerance. As the Arctic warms, and this and other species experience increased periods of heat stress, a limited capacity for evaporative cooling may force birds to increasingly rely on behavioral thermoregulation, such as minimizing activity, at the expense of diminished performance or reproductive investment.

## Graphical abstract

We tested the hypothesis that the evolved physiological mechanisms promoting cold tolerance in snow buntings (*Plectrophenax nivalis*) would result in a limited capacity to tolerate moderate heat. We show that buntings experiencing increasing air temperature begin to evaporate water at temperatures much lower than what is typically seen in more heat‐tolerant songbirds. Most importantly, buntings were extremely inefficient at evaporative cooling, with 95% of heat‐stressed buntings unable to dissipate an amount of heat equivalent to their own metabolic heat production

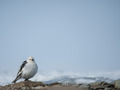

## 1 INTRODUCTION

The Arctic is warming faster than the global average (Overland et al., 2019), impacting both the flora and fauna (CAFF, 2013). Among Arctic birds, climate change has already impacted populations through habitat loss (Gilg et al., 2016), phenological shifts (Moe et al., 2009), and increased predation risk (Smith et al., 2010). However, the direct costs of increasing ambient temperature on the thermoregulatory demands of Arctic birds have garnered far less attention (Gaston et al., 2002). This is particularly concerning given that Arctic species are highly adapted to cold environments and the physiological mechanisms enhancing cold tolerance may increase thermal sensitivity to, and reduce thermoregulatory capacity at, warmer temperatures (Angilletta et al., 2010; Boyles et al., 2011). For example, thick‐billed murres (*Uria lomvia*) can die during incubation when exposed to full sun and daily maximum air temperature of only 16°C (Gaston & Elliott, 2013; Gaston et al., 2002). The paucity of information on Arctic birds’ capacity to physiologically tolerate warmer temperatures is a major impediment to predicting community responses to climate change, especially given their potentially limited ability to cope with heat.

Quantifying avian physiological capacity to tolerate warmer temperatures is fundamental for predicting the impact of climate change on avian biodiversity across biomes (Albright et al., 2017; McKechnie & Wolf, 2010). For example, avian sensitivity to heat has helped link population declines in Mojave Desert birds to climate change‐driven increases in evaporative cooling demands (Riddell et al., 2019). In Australia, intense heat waves have exceeded species' physiological heat tolerance limits, resulting in mass die‐off events (McKechnie et al., 2012). While heat waves produce the most dramatic effects, recent evidence suggests that the fitness costs of climate change will likely occur via sublethal effects from chronic exposure to warmer temperatures (Conradie et al., 2019; Gardner et al., 2015). Specifically, rising temperatures may force birds to increase thermoregulatory behaviors (e.g., shade seeking) at the expense of other essential activities (e.g., foraging; Oswald et al., 2019; Silva et al., 2015). These trade‐offs could significantly affect body condition and fitness of adults and/or nestlings (Cunningham et al., 2013; du Plessis et al., 2012; Van de Ven et al., 2019). Currently, investigations of behavioral trade‐offs and consequent fitness costs from chronic exposure to sublethal temperatures come mostly from arid bird communities, despite recent evidence suggesting that temperate species may also face thermal constraints to increasingly warm temperatures (e.g., Milne et al., 2015; Nilsson & Nord, 2018; Nord & Nilsson, 2019; Oswald et al., 2018; Tapper et al., 2020). However, we also require data on the heat tolerance capacity of Arctic birds as this is necessary to inform predictions of when and how increasing temperatures could impose thermal constraints that would force behavioral trade‐offs and, ultimately, impact fitness.

To address this issue, we investigated the heat tolerance and evaporative cooling capacity of a free‐living population of snow buntings (*Plectrophenax nivalis*) in the Canadian High Arctic. Buntings are a cold specialized, circumpolar migrant passerine that spends most of its life in cold environments. Indeed, buntings overwinter in snowy climates before migrating north through equally harsh conditions during the spring, only to arrive on their Arctic breeding grounds when T_a_ remains below freezing (Meltofte, 1983; Snell et al., 2018). Consequently, buntings have evolved physiological traits to withstand extreme cold (Scholander et al., 1950) and can tolerate experimental temperatures as low as −90°C (Le Pogam et al., 2020). Only later in the season, near the breeding period, do buntings regularly experience maximum temperatures above freezing (Meltofte, 1983). Importantly, in many northern locations temperatures are increasing during the breeding months (Zhang et al., 2019), and this period coincides with the energetically demanding behavior of feeding nestlings. Hence, snow buntings offer an excellent opportunity to examine whether cold‐adapted birds have a limited capacity to physiologically tolerate increasing exposure to moderate heat.

We examined heat tolerance in buntings by measuring responses in body temperature (*T*_b_), resting metabolic rate (RMR), and rates of evaporative water loss (EWL) of individuals exposed to increasing air temperature (*T*_a_). For each physiological trait, we determined the onset of heat stress by identifying *T*_a_ inflection points, which represent the *T*_a_ where the trait starts to change abruptly due to increasing heat. We predicted that relative to previously studied, noncold specialist songbirds, buntings would display inflection points at lower *T*_a_, resulting in an inability to tolerate maximum *T*_a_ typically observed in more heat‐tolerant species.

## 2 MATERIALS AND METHODS

### 2.1 Study species and site

We studied snow buntings between May and July 2018 (*n* = 22 birds) and 2019 (*n* = 20) at Alert, Nunavut, Canada (82°30′05″N, 62°20′20″W). We used walk‐in traps baited with mixed seeds, or potter traps paired with a decoy bird and playback of a bunting call, to capture birds. Additionally, we captured nestling‐provisioning adults at nest entrances after they entered a nest, in which case only one adult was captured at a time, allowing the other parent to continue provisioning. Once captured, we transferred buntings to our field laboratory where they were held in indoor cages (76 cm W × 46 cm D × 45 cm H) for an average of 1.9 ± 2.2 days before respirometry measurements. Birds were maintained on a diet of mixed seeds supplemented with mealworms. All birds were weighed at capture and before respirometry measurements. Average (±*SD*) body mass (*M*_b_) at capture was 33.7 ± 2.5 g and before respirometry measurements was 31.0 ± 2.1 g.

### 2.2 Temperature and gas exchange measurements

We recorded T_b_ using two methods. In 2018, we measured core *T*_b_ using a type‐T thermocouple inserted ≈1 cm into the cloaca. Prior to insertion, the thermocouple tip was lubricated with Vaseline. The thermocouple wire was secured in place on the underside of the tail with masking tape. This technique has been extensively used (see Milne et al., 2015; Prinzinger et al., 1991) and birds calmed down within minutes after insertion. The thermocouple was connected to a Sable Systems thermocouple meter (model TC‐2000, Las Vegas, NV, USA) that measured *T*_b_ every second. In 2019, we measured subcutaneous *T*_b_ using a temperature‐sensitive passive integrated transponder (PIT) tag (Biomark) implanted subcutaneously into the right flank under the wing (Nord et al., 2016). As with the thermocouple, birds calmed down within minutes after implantation. We recorded *T*_b_ every 20 s using a portable transceiver system (model HPR Plus, Biomark) connected to an external racket antenna placed beside the metabolic chamber. After the 2019 field season, we compared a subset of 30 PIT tags in a circulating water bath against a type‐T thermocouple and thermocouple meter (model TC‐2000, Sable Systems). Thermocouple and PIT tag readings were recorded at water temperatures between 40 and 46°C at 2°C increments. On average, PIT tag readings deviated from the thermocouple by 0.2 ± 0.1°C.

To determine RMR and rates of EWL, we measured oxygen consumption (ml/min) and water vapor pressure (WVP; kPa), respectively, using flow‐through respirometry. We placed buntings individually inside a 2.6‐L plastic metabolic chamber fitted with a mesh base with spaces large enough for urine and feces to fall through and into a reservoir of mineral oil. The oil prevented evaporation from excrement affecting WVP measurements. We placed the metabolic chamber inside a temperature‐controlled cabinet fitted with a Peltier heating unit (model T35 DC‐S, Mobicool International). We monitored and regulated the *T*_a_ inside the cabinet using an Omega benchtop controller (model CSi32T). We measured T_a_ inside the metabolic chamber with a type T thermocouple secured underneath the lid and connected to the thermocouple meter.

We pushed atmospheric air through the metabolic chamber with an aquarium air pump (model AAPA15L, Active AQUA). Atmospheric air first passed through columns of silica gel, soda lime, and drierite connected in series to scrub the airstream of water vapor and CO_2_. Once scrubbed, the airstream was split into a baseline channel, which went directly to the analyzers and another channel, which flowed toward the metabolic chamber. We controlled the flow rate of air entering the metabolic chamber with an Omega mass flow controller (model FMA5418A), calibrated against a soap bubble meter (Bubble‐O‐Meter). We maintained flow rates at 2,000 ml/min during the 2018 field season and at 2,500 ml/min in the 2019 field season. These flow rates produced chamber dew points ranging from −20.0 to 9.5°C (maximum absolute humidity = 8.2 g/m^3^ at *T*_a_ = 42.2°C) and the system reached 95% of its final value after either 3.1 or 3.9 min, based on equation 8.1 of Lighton (2019).

We subsampled the incurrent baseline and excurrent chamber airstreams by manually switching between them using a MUX Flow‐Multiplexer (Sable Systems). Subsampled air first passed through a relative humidity and dew point analyzer (model RH‐300; Sable Systems) for the measurement of WVP. The airstream was then scrubbed of water vapor and CO_2_ before entering a Foxbox field gas analysis system (Sable Systems) for the measurement of oxygen consumption. We digitized voltage outputs from all the analyzers using a Sable Systems Universal Interface (model UI‐2) and logged analyzer outputs at a sampling rate of 1 s with Expedata software (Sable Systems).

### 2.3 Experimental protocol

We performed respirometry measurements between 10:00 and 00:00 hr depending on the time of capture and the need to process birds as quickly as possible. Once placed inside the metabolic chamber, we gave buntings a 30‐min habituation period to acclimate to the chamber before being exposed to a ramped *T*_a_ profile. In 2018, we started birds at *T*_a_ ≈ 25°C with an increase to 30°C and then at 2°C increments. In 2019, we started measurements at *T*_a_ ≈ 30°C with subsequent increases at 2°C increments. We began measurements at 30°C in 2019 as our primary goal during this second field season was to increase sample sizes at higher *T*_a_, and this *T*_a_ typically did not invoke heat stress in 2018. Once chamber *T*_a_ stabilized, we recorded data on buntings for 10–20 min before increasing *T*_a_. A 10‐min baseline was recorded at the beginning and end of each run to control for analyzer drift. We continuously monitored the behavior of each focal bird using a SmoTecQ dome infrared camera (model DF‐3500‐AHD 1080P) and video capture software (ArcSoft ShowBiz, v. 3.5.15.68). We ended runs if buntings displayed continuous escape behavior (e.g., pecking at the walls of the chamber or jumping), or a *T*_b_ ≥ 45°C. After each run, we immediately measured the bird's mass, provided them with fresh water, and returned them to their cage for release.

### 2.4 Data analyses

We first corrected the oxygen consumption and WVP traces for drift and time lag using the appropriate operations in Expedata. At each *T*_a_, we measured resting values of oxygen consumption, WVP, and *T*_b_ using the mean of the most stable 5‐min period from the oxygen consumption trace. We did not include any data from birds that did not remain calm for at least 5 min at a given *T*_a_. We calculated rates of oxygen consumption using equation 10.1 of Lighton (2019). To transform oxygen consumption into RMR (Watts [W]), we used equation 9.13 of Lighton (2019) to derive energy equivalents (J/mlO_2_) assuming a respiratory quotient (RQ) of 0.71. However, in some cases (12%), birds were not fasted for more than 62 min (mean retention time for a 31 g bird; Karasov, 1990), and for these birds, we assumed an RQ of 0.80 (Lighton, 2019). We calculated rates of EWL (mg/min) by converting WVP into water vapor density and then multiplying by the incurrent flow rate. We converted rates of EWL into evaporative heat loss (EHL; W) assuming 2.406 J/mgH_2_O. We determined how efficient buntings were at dissipating body heat by calculating their evaporative cooling efficiency, which represents the ratio between EHL and metabolic heat production (EHL/MHP). Higher EHL/MHP values indicate greater evaporative cooling efficiency (Lasiewski et al., 1966).

We performed all statistical analyses in R 4.0.0 (R Core Team, 2020), and all values reported are means ± standard deviation (*SD*), unless noted otherwise. During our initial analyses, we found that *T*_b_ varied considerably at a given *T*_a_ depending on measuring technique (Supporting information). However, because recent heat tolerance investigations measured core *T*_b_ (e.g., McKechnie et al., 2017; Smith et al., 2017; Whitfield et al., 2015), and thus to facilitate comparisons, we decided to only report our core *T*_b_ values measured in the cloaca.

We first located an inflection point for each response variable, namely *T*_b_, RMR, EWL, and EHL/MHP, by fitting a piecewise linear regression model to the data with all birds combined using the *SiZer* package (Sonderegger, 2020). For each response variable, we subsequently fitted a linear mixed‐effect model to the data above the inflection point using the *lme4* package (Bates et al., 2015). Each mixed‐effect model included *T*_a_ and *M*_b_ as continuous predictors. We included bird identity as a random intercept in all our models to account for repeated measurements within the same bird. We built a global model with all predictors and their two‐way interaction (i.e., *T*_a_:*M*_b_). We performed model selection on the global models using the “dredge” function in the *MuMIn* package (Bartoń, 2020). Models with an Akaike information criterion adjusted for small sample sizes (AIC_c_) less than 8 (i.e., ∆AIC_c_ < 8) were considered to fit the data equally well (Burnham et al., 2011). Additionally, we used the model weights for each model to assess their relative strength of support, with models having a weight > 0.90 considered to exhibit overwhelming support as the best approximating model relative to all the other candidate models (Grueber et al., 2011). We further explored each top model and report the parameter estimates and accompanying standard errors (*β* ± *SE*), 95% confidence intervals (95% CI), and *t*‐values for each fixed effect in the model.

We assessed the overall fit of the global and top candidate models by visually inspecting the residuals for normality and homogeneity. Additionally, we tested for outliers in all the models by calculating a Cook's distance value for every bird using the *influence*.*ME* package (Nieuwenhuis et al., 2012). We considered birds with a Cook's distance value > 1 as highly influential on the parameter estimates (Logan, 2010). One model had Cook's distance values > 1, and instead of removing these values from the data set, we fitted a robust mixed‐effect model to the data using the *robustlmm* package (Koller, 2016). All figures were made using *ggplot2* (Wickham, 2016), and the 95% CI around the regression predictions was calculated in *ggeffects* (Lüdecke, 2018).

## 3 RESULTS

### 3.1 Body temperature

**FIGURE 1:**
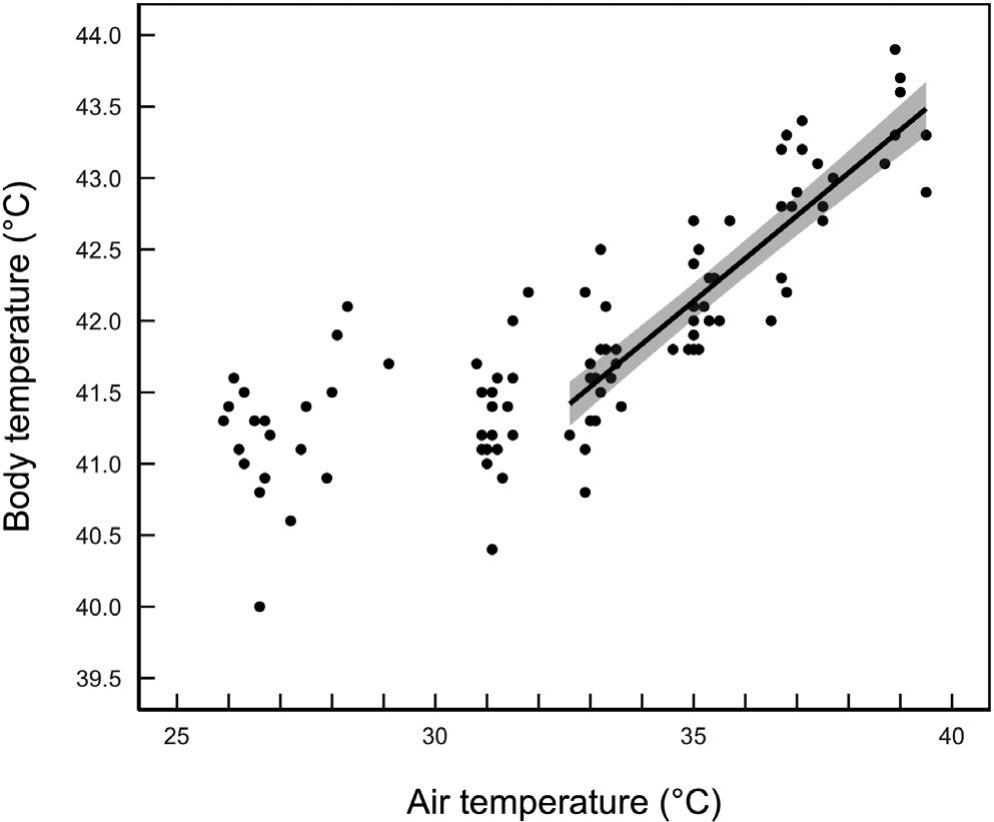
The relationship between core body temperature (*T*_b_) measured in the cloaca and air temperature (*T*_a_). The regression line represents the slope from a linear mixed‐effects model of *T*_b_ regressed against *T*_a_ above the inflection point (32.6°C). The shaded area represents the 95% confidence intervals around the predicted values

Snow bunting body temperature showed an inflection point at *T*_a_ = 32.6°C (95% CI = 31.0–34.4°C; Figure 1). Above the inflection point, the top candidate model fitted to the data included only *T*_a_ and was much better supported than models including *M*_b_ or the *T*_a_:*M*_b_ interaction (Table 1). Body temperature had a positive linear relationship with T_a_ above the inflection point (*n* = 21, Figure 1; Table 2). Body temperature increased from 41.3 ± 0.2°C (*n* = 6) at *T*_a_ ≈ 26°C to 43.5 ± 0.3°C (*n* = 5) at *T*_a_ ≈ 39.0°C.

**TABLE 1:** Top candidate models after model selection with an Akaike information criterion adjusted for small sample size less than 8 (i.e., ∆AICc < 8)

Variable	Top models^a^	logLik	AICc	∆AICc	Model weight
*T*_b_	*T*_a_	−17.759	44.29	0.000	0.940
	*T*_a_ + *M*_b_	−19.322	49.82	5.532	0.059
RMR	*T*_a_	82.842	−157.39	0.000	0.965
	*T*_a_ + *M*_b_	80.484	−150.53	6.865	0.031
EWL	*T*_a_	59.491	−110.33	0.000	0.991
EHL/MHP	*T*_a_	13.147	−17.04	0.000	0.641
	*T*_a_ + *M*_b_	13.910	−15.88	1.161	0.359

Note

Models reflect data above the inflection points. Model selection was performed on four separate global models, each with a different response variable, namely body temperature (*T*_b_), resting metabolic rate (RMR), evaporative water loss (EWL), and the ratio of evaporative heat loss to metabolic heat production (i.e., evaporative cooling efficiency; EHL/MHP). Model fixed effects were air temperature (*T*_a_) and body mass (*M*_b_). Models with a weight > 0.90 were considered to have overwhelming support.

^a^ Global model included *T*_a_ + *M*_b_ + *T*_a_:*M*_b_.

**TABLE 2:** Parameter estimates (*β* ± standard error) from the top linear mixed‐effects models (see Table 1) explaining variation in body temperature (*T*_b_), resting metabolic rate (RMR), rates of evaporative water loss rate (EWL), and the ratio of evaporative heat loss to metabolic heat production (i.e., evaporative cooling efficiency; EHL/MHP)

Variable	*T*_a_ inflection	*β* ± *SE*	95% CI	*t*‐Value
*T*_b_ (°C)	32.6°C	–	–	–
Intercept	–	31.66 ± 0.60	30.47 to 32.84	52.49
*T*_a_	–	0.299 ± 0.017	0.266 to 0.333	17.62
RMR (Watts)	29.8°C	–	–	–
Intercept	–	0.208 ± 0.103	0.007 to 0.410	2.03
*T*_a_	–	0.014 ± 0.003	0.009 to 0.020	5.10
EWL (g/hr)	34.6°C	–	–	–
Intercept	–	−2.00 ± 0.15	−2.30 to −1.70	−13.22
*T*_a_	–	0.068 ± 0.004	0.060 to 0.076	16.66
EHL/MHP	36.7°C	–	–	–
Intercept	–	−2.05 ± 0.32	−2.68 to −1.43	−6.43
*T*_a_	–	0.068 ± 0.008	0.052 to 0.084	8.24

Note

Parameter estimates are derived from models fitted to the data above the calculated air temperature inflection points (*T*_a_ inflection). The 95% confidence intervals (95% CI) and *t*‐values from the models are included.

### 3.2 Resting metabolic rate

**FIGURE 2:**
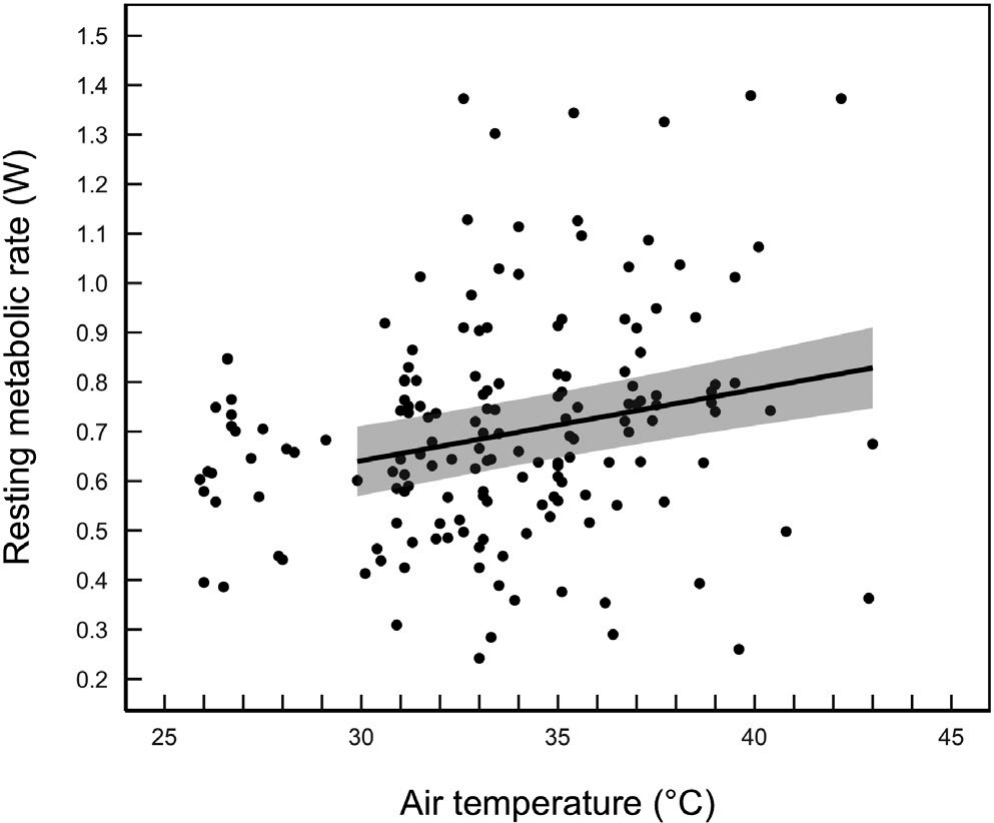
The relationship between resting metabolic rate (RMR) and air temperature (*T*_a_) in snow buntings. The regression line represents the slope from a linear mixed‐effects model of RMR against *T*_a_ fitted to data above the inflection point (29.8°C). The shaded area represents the 95% confidence intervals around the predicted values

Resting metabolic rate had an inflection point at *T*_a_ = 29.8°C (95% CI = 27.9–42.2; Figure 2). Above the inflection point, the top candidate model included only *T*_a_ and had overwhelming support compared with the other candidate models (Table 1). Above the inflection point, RMR increased gradually with *T*_a_ (Table 2, Figure 2). Over the range of temperatures measured, RMR displayed a 1.4‐fold increase, from 0.588 ± 0.105 W at *T*_a_ ≈ 26 to 0.804 ± 0.517 W at *T*_a_ ≈ 43°C.

### 3.3 Evaporative water loss

**FIGURE 3:**
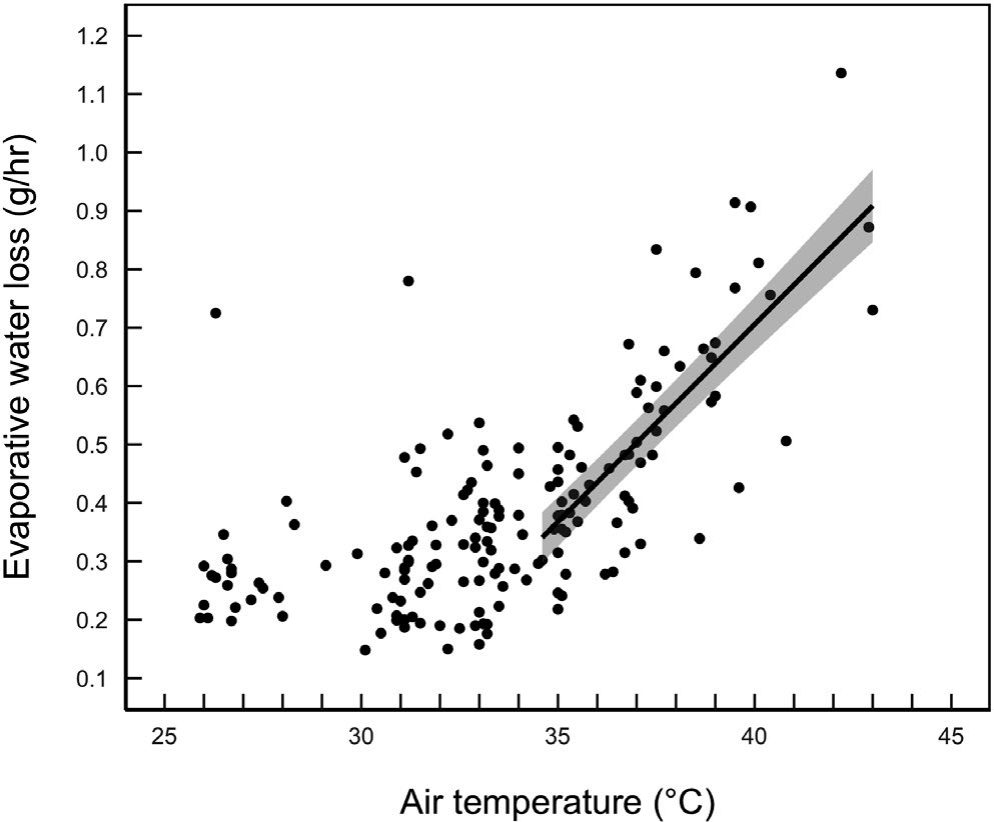
The relationship between rates of evaporative water loss (EWL) and air temperature (*T*_a_) in snow buntings. The regression line represents the slope from a linear mixed‐effects model of EWL against *T*_a_ fitted to the data above the inflection point (34.6°C). The shaded area represents the 95% confidence intervals around the predicted values

On average, buntings began panting at *T*_a_ = 33.2 ± 1.7°C in 2018 and 33.6 ± 1.8 in 2019. The average T_b_ at the start of panting was 42.0 ± 0.8°C. The onset of panting coincided with the EWL inflection point at *T*_a_ = 34.6°C (95% CI = 31.1–36.2; Figure 3). Above the inflection point, the top candidate model only included *T*_a_ and had overwhelming support relative to the other candidate models (Table 1). Above the inflection point, EWL displayed a positive linear relationship with *T*_a_ (Table 2 and Figure 3). Buntings increased their rate of EWL 2.9‐fold relative to baseline rates at *T*_a_ ≈ 26°C, reaching a maximum average rate of EWL = 0.913 ± 0.206 g/hr at *T*_a_ ≈ 43°C.

### 3.4 Evaporative cooling efficiency

**FIGURE 4:**
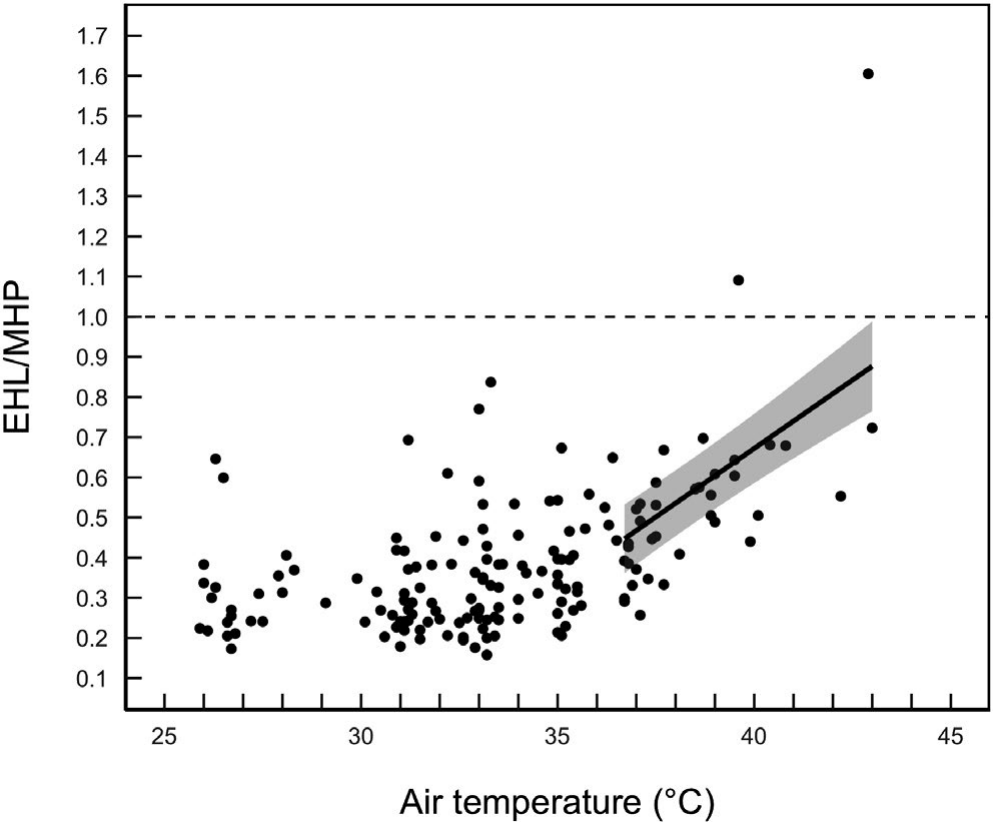
The relationship between the ratio of evaporative heat loss (EHL) to metabolic heat production (MHP) and air temperature (*T*_a_) in snow buntings. The black regression line represents the slope from a linear mixed‐effects model of EHL/MHP against *T*_a_ above the inflection point (36.7°C). The shaded area represents the 95% confidence intervals around the predicted values. The horizontal dashed line represents the ratio when birds are able to evaporatively dissipate 100% of their MHP

Buntings exhibited an EHL/MHP inflection point at *T*_a_ = 36.7 (95% CI = 31.0–42.3°C; Figure 4). Above the inflection point, the top model explaining variation in EHL/MHP only included *T*_a_ (Table 1). However, there was some support for the second model, which included *M*_b_ and *T*_a_ (Table 1). There was a positive linear relationship between EHL/MHP and *T*_a_ above the inflection point (Table 2, Figure 4). Only two birds exceeded an EHL/MHP of 1.0 (Figure 4), indicating that most buntings were always producing more heat metabolically than they were losing evaporatively. Moreover, only 5 birds (i.e., 12%) had EHL/MHP values exceeding 0.70 (Figure 4), highlighting that buntings were extremely inefficient at dissipating heat evaporatively. The magnitude of increase in EHL/MHP was 2.8‐fold, increasing from an average of 0.348 ± 0.144 at *T*_a_ ≈ 26.0°C up to an average of 0.960 ± 0.565 at *T*_a_ ≈ 43.0°C.

## 4 DISCUSSION

Our goal was to examine the heat tolerance and evaporative cooling capacity of an Arctic songbird. As predicted, snow buntings increased their resting metabolic rate and rate of evaporative water loss at air temperatures well below their body temperature, indicating an early onset of heat stress. Moreover, buntings tolerated consistently lower ambient temperatures than previously studied heat‐tolerant songbirds. Thus, the physiological mechanisms permitting buntings’ extreme cold tolerance seem to adversely affect their heat tolerance. Indeed, heat stressed buntings exhibited low evaporative cooling efficiencies, with most individuals unable to evaporatively dissipate more than 70% of their metabolic heat production. Thus, we predict buntings will become increasingly challenged to physiologically dissipate body heat as the Arctic warms. Indeed, preliminary data collected within buntings’ Arctic breeding range show that maximum environmental operative temperatures (Bakken, 1976) can exceed 30°C (R. S. O’Connor, O. P. Love, K. H. Elliott, & F. Vézina unpublished data). Below, we compare our findings with recent heat tolerance studies on songbirds and conclude by discussing the ecological implications of our findings.

### 4.1 Body temperature

When exposed to increasing heat loads, birds often allow *T*_b_ to increase with *T*_a_ (i.e., facultative hyperthermia; Gerson et al., 2019; Tieleman & Williams, 1999). Buntings displayed increases in *T*_b_ starting at *T*_a_ of 32.6°C, which is within the range reported for 26 desert and nondesert species (Tieleman & Williams, 1999). Similarly, the rate at which bunting *T*_b_ changed with *T*_a_ is comparable to that found in other passerines (Czenze et al., 2020; McKechnie et al., 2017; Weathers, 1981). Thus, body temperature patterns of buntings under heat stress appear broadly similar to those of other avian species.

**FIGURE 5:**
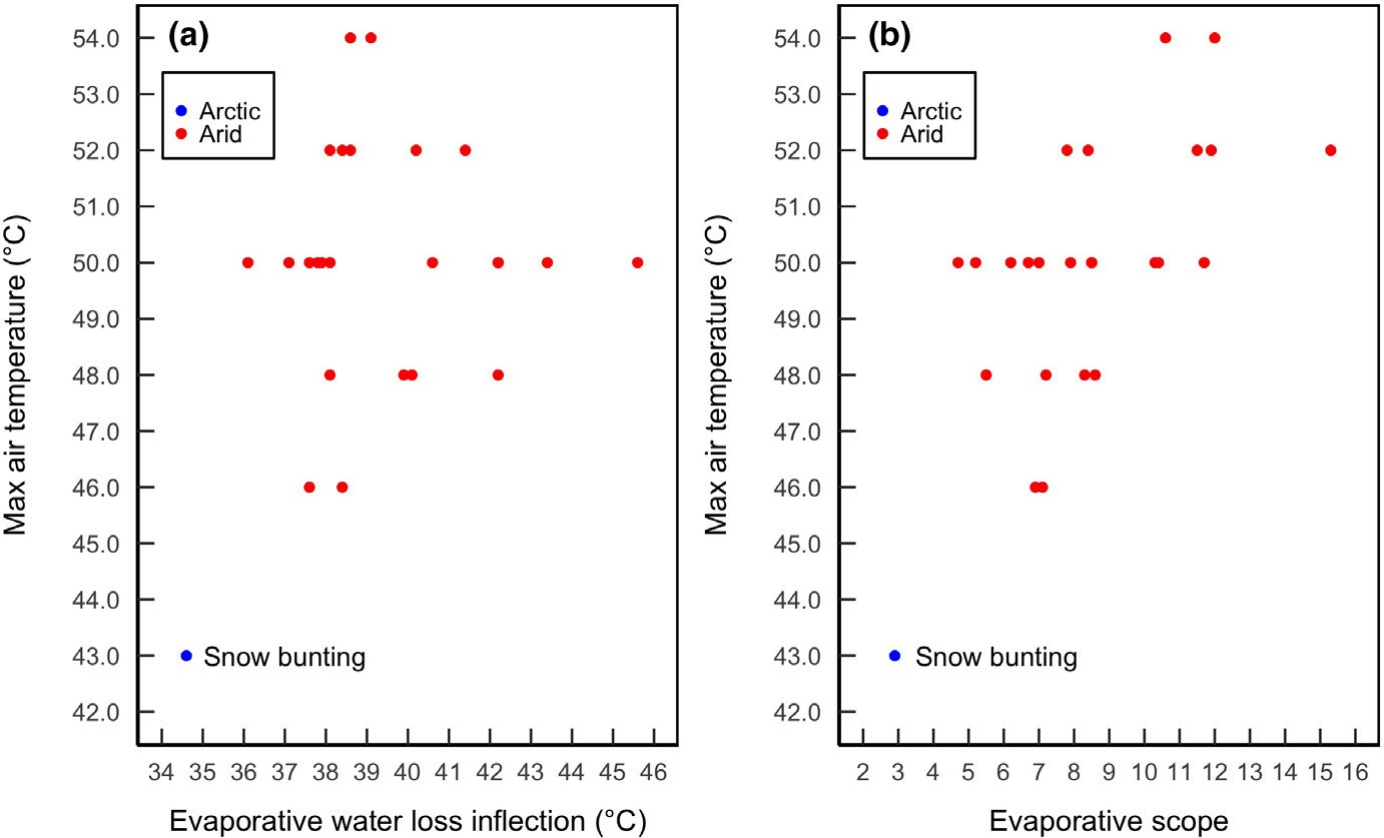
Maximum air temperature tolerated as a function of (a) evaporative water loss inflection point and (b) average evaporative scope among 24 arid‐zone passerines and Arctic snow buntings. Evaporative scope represents the ratio between the maximum rate of EWL and the minimum rate of EWL (sensu Czenze et al., 2020). Arid species data are from McKechnie et al. (2017), Smith et al. (2017), and Czenze et al. (2020)

The maximum *T*_a_ at which birds can defend a sublethal *T*_b_ frequently correlates with the climate of origin, with species from warmer, more arid environments generally tolerating hotter temperatures (e.g., Hudson & Kimzey, 1966; McKechnie & Wolf, 2019; Noakes et al., 2016; Noakes et al., 2016; Tieleman et al., 2002). Our data support this trend, as the maximum *T*_a_ at which buntings regulated *T*_b_ was lower than for 24 arid‐zone passerines (Figure 5). This suggests that the physiological mechanisms enhancing heat tolerance are less pronounced in buntings and they may need to adjust their behavior at much lower environmental temperatures to avoid overheating.

### 4.2 Resting metabolic rate

Resting energy expenditure tends to vary inversely with habitat temperature, wherein birds from warmer environments have lower basal rates of heat production than species from colder environments (Jetz et al., 2008; Tieleman & Williams, 2000; Weathers, 1979; White et al., 2007). We found that at *T*_a_ ≈ 26°C (i.e., within buntings’ thermoneutral zone; Scholander et al., 1950), mean RMR was 31% higher than the average minimum RMR reported in an arid population of similarly sized red‐eyed bulbuls (*Pycnonotus nigricans,* 30.1 g; Czenze et al., 2020). Furthermore, the basal metabolic rate (BMR) for 138 buntings (mean *M*_b_ ≈ 33.1 g) measured at Alert was 0.563 W (unpublished data from authors), a value 154% of the prediction for a passerine of its size (Londoño et al., 2015). Hence, our findings are consistent with the trend that species from colder environments have higher rates of resting energy expenditure. Although a higher metabolic rate is likely advantageous for life in the cold, it presumably becomes a hindrance in warmer conditions because the higher metabolic heat production will lead to a greater total heat load that must be dissipated (Bartholomew et al., 1962).

Buntings displayed an upper critical temperature (*T*_uc_) in RMR at *T*_a_ of 29.8°C, which is lower than the *T*_uc_ values reported for some arid and mesic passerines, ranging from 33.9 to 44.9°C (McKechnie et al., 2017; Smith et al., 2017; Tieleman et al., 2002; Weathers, 1981). This is consistent with our prediction that buntings would exhibit signs of heat stress at lower *T*_a_ relative to noncold specialist songbirds. Lower *T*_uc_ values are also apparent in species from cooler habitats when directly compared to closely related species from warmer regions (e.g., Hayworth & Weathers, 1984; Tieleman et al., 2002; Weathers & van Riper, 1982). A lower *T*_uc_ should limit a species heat tolerance because the early contribution of metabolic heat above basal levels will add to the total heat load that must be dissipated at a given *T*_a_.

The slope of RMR above the *T*_uc_ represents the cost of thermoregulation (Weathers, 1981). Shallower slopes (i.e., less heat produced per unit of increase in *T*_a_) are expected in more heat‐tolerant species (Cooper & Gessaman, 2004) because the minimization of metabolic heat production above basal levels will lower an individual's total heat load (Bartholomew et al., 1962). Our results do not support this expectation. For example, the slope of RMR against *T*_a_ for buntings (0.014 W/°C) is 43% shallower than the slope for the similarly sized, more heat‐tolerant red‐eyed bulbul (Czenze et al., 2020). Furthermore, buntings’ fractional increase in metabolic rate (i.e., maximum RMR/minimum RMR) of 1.4 is identical to the average fractional increase reported for six Sonoran Desert songbirds (Smith et al., 2017). Hence, although buntings appear to have high resting energy expenditures and a low *T*_uc_, both of which should adversely affect their heat tolerance by contributing to a greater total heat load at high *T*_a_, the incurred metabolic cost from panting does not seem to exceed that of more heat‐tolerant species who also use panting.

### 4.3 Evaporative water loss

Buntings started panting at a low mean *T*_a_ of 33.2°C, with a subsequent increase in EWL at *T*_a_ of 34.6°C. Milne et al. (2015) reported a panting *T*_a_ value of 33.6°C in a population of cape rockjumpers (*Chaetops frenatus*) inhabiting the cool, high‐altitude regions of South Africa. Together, these findings suggest that species originating from cooler regions may experience heat stress at lower *T*_a_. These patterns starkly contrast those of more heat‐tolerant passerines. For example, among 17 arid‐zone passerines, the lowest average *T*_a_ at the onset of panting was 38.0°C (Czenze et al., 2020). Moreover, the EWL inflection point and evaporative scope (i.e., max EWL/min EWL; sensu Czenze et al., 2020) were consistently lower for buntings than for 24 arid‐zone passerines (Figure 5). Recently, Czenze et al. (2020) observed that heat tolerance limits among arid‐zone passerines correlated with higher evaporative scopes. Our data conform to this pattern, as buntings displayed a low evaporative scope and a correspondingly low maximum *T*_a_ (Figure 5). Buntings’ low evaporative scope presumably contributed, in part, to their limited heat tolerance capacity by constraining the amount of heat they could dissipate evaporatively.

### 4.4 Evaporative cooling efficiency

In contrast to more heat‐tolerant passerines (McKechnie et al., 2017; Whitfield et al., 2015), buntings exhibited generally low evaporative cooling efficiencies, with only two individuals evaporatively dissipating more heat than produced metabolically (cooling efficiencies of 1.09 and 1.61). Moreover, 88% of buntings could not dissipate more than 70% of their own metabolic heat through evaporation, further exemplifying how inefficient buntings are at dissipating body heat. Interestingly, Oswald et al. (2018) measured cape rockjumpers up to *T*_a_ of 42°C and found that no birds exceeded an EHL/MHP value of 1, further suggesting that species that regularly inhabit cooler climates are potentially more vulnerable to moderate heat. The inability to efficiently dissipate their own metabolic heat production must severely limit buntings’ capacity to tolerate moderately high temperatures.

### 4.5 Conclusions and ecological implications

To our knowledge, this is the first study investigating how an Arctic songbird responds physiologically to warmer temperatures. We found that buntings had a limited capacity to tolerate increasing temperatures, manifested through several interacting physiological traits: (a) high rates of resting energy expenditure (e.g., basal heat production), (b) early onset of increases in resting metabolic rate and evaporative water loss under warming conditions, and (c) a limited evaporative scope. These factors culminated in buntings having generally low evaporative cooling efficiencies. Indeed, most buntings were incapable of evaporatively dissipating an amount of heat equivalent to their own metabolic heat production. These findings suggest that the physiological mechanisms permitting extreme cold tolerance in buntings, and possibly Arctic birds generally, inhibit their capacity to tolerate even moderately warm conditions. By the late 21st century, annual mean temperature across Canada could increase by more than 6°C, with the greatest warming occurring in northern regions (Zhang et al., 2019). Under this scenario, buntings will increasingly encounter environmental temperatures exceeding their physiological thresholds for heat stress. Given buntings’ extreme inefficiency for evaporative cooling, we predict they will increasingly rely on behavioral strategies for thermoregulation, which can interfere with provisioning rates and foraging efficiency (Cunningham et al., 2013; du Plessis et al., 2012). Ultimately, we expect behavioral trade‐offs to significantly impact performance during the summer breeding season, creating another example of sublethal effects of warming reducing avian fitness (Conradie et al., 2019). Hence, we argue that Arctic birds will not be exempt from thermal constraints due to increasing temperatures.

A major hurdle for leveraging thermal physiology data to predict climate change responses is extrapolating laboratory data to field scenarios (Bakken, 1976). One critical issue is that laboratory data, like ours, are collected on resting birds, whereas free‐living individuals are active and have higher sustained metabolic rates. This means that active birds will have a greater total heat load at any given temperature and should thus experience heat stress at lower environmental temperatures than predicted for resting birds. Recently, Rezende and Bacigalupe (2015) proposed a novel approach (i.e., thermoregulatory polygon) for combining the metabolic contribution of an active animal with standard respirometry variables to predict the range of conditions under which passive thermoregulation is possible. Using this framework, we estimate that buntings operating at 4 times BMR could maintain a constant T_b_ up to environmental temperatures of 22°C, above which they would either have to begin evaporative cooling or reduce activity to avoid hyperthermia. Hence, an active bunting would have to increase its rate of EWL at an environmental temperature of 11.2°C below the EWL inflection point reported here for resting birds. Importantly, buntings already experience environmental temperatures exceeding 22°C across their breeding range (unpublished data from authors). Thus, although we expect bunting populations to increasingly experience thermal constraints in the future, it is possible that sublethal effects of Arctic warming occurring via thermal trade‐offs (e.g., increasing thermoregulatory behaviors at the expense of nestling provisioning and development; Cunningham et al., 2013) are already occurring in these cold specialists, and possibly in cold adapted Arctic species generally.

## CONFLICT OF INTEREST

None declared.

## AUTHOR CONTRIBUTIONS

**Ryan S. O'Connor:** Conceptualization (lead); Data curation (lead); Formal analysis (lead); Writing‐original draft (equal). **Audrey Le Pogam:** Investigation (equal); Writing‐review & editing (equal). **Kevin G. Young:** Investigation (equal); Writing‐review & editing (equal). **Francis Robitaille:** Investigation (equal); Writing‐review & editing (equal). **Emily S. Choy:** Writing‐review & editing (equal). **Oliver P. Love:** Conceptualization (equal); Writing‐review & editing (equal). **Kyle H. Elliott:** Conceptualization (equal); Writing‐review & editing (equal). **Anna L. Hargreaves:** Conceptualization (equal); Writing‐review & editing (equal). **Dominique Berteaux:** Writing‐review & editing (equal). **Andrew Tam:** Writing‐review & editing (equal). **François Vézina:** Conceptualization (equal); Writing‐original draft (equal).

## Supporting information

Supplementary Material

## Data Availability

Respirometry data analyzed for this study are available from the Dryad Digital Repository (https://doi.org/10.5061/dryad.4mw6m908g).
