# Supplementary material for: Limited heat tolerance in an Arctic passerine: Thermoregulatory implications for cold‐specialized birds in a rapidly warming world
Source: Ecol Evol. 2021 Jan 17;11(4):1609–19. doi: 10.1002/ece3.7141 (PMC7882984; doi:10.1002/ece3.7141)
Supplement: Supplementary file 1 — Supplementary Material [file ECE3-11-1609-s001.docx]

**Supporting information**

*Cloacal T_b_ (T_b_ – cloaca) versus subcutaneous T_b_ (T_b_ – PIT)*

Cloacal T_b_ had a lower inflection point (32.6 °C, 95% CI = 31.0 – 34.4 °C) than T_b_ - PIT (36.4 °C, 95% CI = 33.5 – 39.6 °C; Figure S1). Above their respective inflection points, the top candidate models fitted to the data only included T_a_ and showed overwhelming support relative to the second-ranked model (Table S1). Cloacal T_b_ and T_b_ – PIT increased linearly with T_a_ at similar rates above their inflection points (Figure S1; Table S2). Across their shared T_a_ range (30.8 – 39.5 °C), T_b_ - cloaca was predominately higher than T_b_ – PIT (Figure S1). Cloacal T_b_ increased by 2.2 °C from an average of 41.3 ± 0.2 °C at T_a_ ≈ 26 °C to an average of 43.5 ± 0.3 °C at T_a_ ≈ 39.0 °C. Similarly, T_b_ – PIT increased by 2.2 °C from 40.4 ± 0.5 °C at T_a_ ≈ 30.0 °C to 42.6 ± 0.5 at T_a_ ≈ 43 °C.

Our data revealed distinct differences in T_b_ among measuring techniques, with cloacal T_b_ generally greater than subcutaneous T_b_ at a given T_a_. Numerous factors may influence an animal’s peripheral temperature (i.e., subcutaneous or skin surface), thereby driving core-to-skin temperature differences, including body size, environmental inputs, and/or peripheral blood flow (Audet and Thomas 1996, Adelman et al. 2010, McCafferty et al. 2015, Rey et al. 2015). Consequently, it has been suggested that all organisms will exhibit regional heterothermy (Angilletta et al. 2010) and our findings may thus represent restricted heat flow between the body core and skin, a finding expected for a cold-specialist species which has evolved to minimize heat loss in harsh environments. O’Connor et al. (*unpublished data*), for example, implanted PIT tags subcutaneously on the nape between the scapulae to measure subcutaneous temperature in captive snow buntings and found core-to-skin differences wherein cloacal temperatures were higher by 0.6 ± 1.0 °C, on average.

**Literature Cited**

Adelman, J. S., Córdoba-Córdoba, S., Spoelstra, K., Wikelski, M. and Hau, M. 2010. Radiotelemetry reveals variation in fever and sickness behaviours with latitude in a free-living passerine. *Funct. Ecol*. 24:813-823. doi:10.1111/j.1365-2435.2010.01702.x.

Angilletta, M. J. Jr., Cooper, B. S., Schuler, M. S. and Boyles, J. G. 2010. The evolution of thermal physiology in endotherms. *Front. Biosci.* E2:861-881.

Audet, D. and Thomas, D. W. 1996. Evaluation of the accuracy of body temperature measurement using external radio transmitters. *Can. J. Zool.* 74:1778-1781.

McCafferty, D. J., Gallon, S. and Nord, A. 2015. Challenges of measuring body temperatures of free-ranging birds and mammals. *Anim. Biotelemetry* 3:33. doi:10.1186/s40317-015-0075-2.

Rey, B., Halsey, L. G., Hetem, R. S., Fuller, A., Mitchell, D. and Rouanet, J. 2015. Estimating resting metabolic rate by biologging core and subcutaneous temperature in a mammal. *Comp. Biochem. Physiol. Part A: Mol. Intergr. Physiol.* 183:72-77. doi:10.1016/j.cbpa.2015.01.012.

**Table S1**. Top candidate models after model selection with an Akaike Information Criterion adjusted for small sample size less than 8 (i.e., ∆AICc < 8). Model selection was performed on two separate global models, each with a different response variable, namely body temperature measured in the cloaca (T_b_ - Cloaca ) and T_b_ measured subcutaneously with passive integrated transponder (PIT) tags (T_b_ - PIT). Models with a weight > 0.90 were considered to have overwhelming support.

| Variable | Model^a^ | logLik | AICc | ∆AICc | Model weight |
| --- | --- | --- | --- | --- | --- |
| T_b_ - Cloaca | T_a_  T_a_ + M_b_ | -17.759  -19.322 | 44.29  49.82 | 0.000  5.532 | 0.940  0.059 |
| T_b_ - PIT | T_a_  T_a_ + M_b_ | -11.69  -12.19 | 34.72  39.84 | 0.000  5.114 | 0.928  0.072 |

^a^ Global model included T_a_ + M_b_ + T_a_:M_b_.

**Table S2**. Parameter estimates (*β* ± standard error) from the top linear mixed-effect models (see Table 1) explaining variation in body temperature (T_b_) measured in the cloaca (T_b_ - Cloaca) and T_b_ measured subcutaneously with PIT tags (T_b_ – PIT). Parameter estimates are derived from models fit to data above the calculated air temperature inflection points (T_a_ inflection). The 95% confidence intervals (95% CI) and t-values from the models are included.

| Variable | T_a_ inflection | *β* ± SE | 95% CI | t-value |
| --- | --- | --- | --- | --- |
| **T_b_ – Cloaca (°C)** | 32.6 °C | - | - | - |
| Intercept | - | 31.66 ± 0.60 | 30.47 – 32.84 | 52.49 |
| T_a_ | - | 0.299 ± 0.017 | 0.266 – 0.333 | 17.62 |
| **T_b_ – PIT (°C)** | 36.4°C | - | - | - |
| Intercept | - | 29.76 ± 1.14 | 27.52 – 32.00 | 26.05 |
| T_a_ | - | 0.308 ± 0.029 | 0.251 – 0.365 | 10.67 |

**Figure S1**. The relationship between core body temperature measured in the cloaca and body temperature measured subcutaneously with passive integrated transponder (PIT) tags with air temperature. Regression lines represent the slopes from separate linear mixed-effects models of T_b_ – Cloaca and T_b_ - PIT regressed against air temperature above their respective inflection points. The shaded areas represent the 95% confidence intervals around the predicted values.
